# Weight Loss Efficacy of Tirzepatide Compared to Placebo or GLP-1 Receptor Agonists in Adults With Obesity or Overweight: A Meta-Analysis of Randomized Controlled Trials With ≥ 20 Weeks Treatment Duration

**DOI:** 10.1155/jobe/3442754

**Published:** 2025-07-24

**Authors:** Alhussain Khawaji, Abdulaziz A. Jaly, Hanan A. Bakri, Renju Ravi, Ahmed Hattan, Abdullah Khawaji, Wael Najmi

**Affiliations:** ^1^Clinical Pharmacy, Al-Iman General Hospital, Riyadh, Saudi Arabia; ^2^Department of Pharmacy, Jazan University Hospital, Jazan, Saudi Arabia; ^3^Clinical Pharmacy, King Fahd Central Hospital, Jazan, Saudi Arabia; ^4^Department of Basic Medical Sciences, Faculty of Medicine, Jazan University, Jazan, Saudi Arabia; ^5^Pharmaceutical Care Services, King Abdullah Bin Abdul-Aziz University Hospital, Riyadh, Saudi Arabia; ^6^Jazan Diabetes and Endocrinology Center, Ministry of Health, Jazan, Saudi Arabia; ^7^Pharmaceutical Care Services, Al-Iman General Hospital, Riyadh, Saudi Arabia

**Keywords:** GLP-1, meta-analysis, obesity, tirzepatide, weight loss

## Abstract

**Introduction:** Tirzepatide, a dual glucose-dependent insulinotropic peptide (GIP) and glucagon-like Peptide 1 (GLP-1) analogue, is a novel medication with comparable pharmacological characteristics and has demonstrated promising weight reduction outcomes in its antidiabetic trials following the approval of liraglutide and semaglutide for long-term weight control. Nonetheless, this efficacy has not been fully explored, so this meta-analysis was aimed to measure the weight loss efficacy and safety of tirzepatide in adults with overweight or obesity.

**Methods:** We searched the PubMed, Cochrane, and Embase databases for RCTs of once-weekly tirzepatide vs. placebo or GLP-1 receptor agonists. We included studies involving adult participants who were overweight or obese despite T2DM or OHA use, with a trial duration of at least 20 weeks. The primary outcomes accounted for the mean difference in weight from baseline in the three doses of tirzepatide compared to placebo and GLP-1 receptor agonists, separately. The secondary outcomes included safety profiles and achievement of categorical weight loss of 5%, 10% and 15%. We performed the statistical analysis on RevMan 5.4, GRADE assessment using GRADEpro GDT and the quality of the included studies assessed using the Cochrane risk-of-bias (Version 2) tool.

**Results:** We identified six RCTs in which the data of 6266 subjects were analysed. Once-weekly doses (5, 10 and 15 mg) of tirzepatide were more effective than placebo and GLP-1 RAs. Also, the proportion of patients achieving categorical weight loss goals was higher in the tirzepatide groups than in others. GRADE assessment also indicated high-certainty evidence for ≥ 15% weight loss with tirzepatide and moderate-to-low certainty for lower thresholds. Gastrointestinal side effects appeared similar between the three doses of tirzepatide and GLP-1 RAs, but they were significantly higher than placebo might impact tolerability for certain patients.

**Conclusion:** A dose-dependent tirzepatide was superior to placebo and GLP-1 RAs in weight reduction. However, the lean mass reduction and tolerability require further investigation.

**Trial Registration:** ClinicalTrials.gov identifier: NCT04255433

## 1. Introduction

Obesity, a body mass index (BMI) of ≥ 30 kg/m^2^, is one of the significant contributors to cardiovascular diseases, diabetes, musculoskeletal disorders and cancers [1, 2]. In the latest WHO report, it was estimated that the prevalence of obesity reached epidemic proportions in 2016, affecting 6.8% of children and 13.1% of adults worldwide [1]. These numbers increased two-fold in more than 70 countries between 1980 and 2015 [2]. Due to the complexity of the disease, the aetiology of obesity can be attributed to physiological, genetic and environmental factors [2, 3]. The current understanding of the physiological property of obesity is based on the fact that fat storage is caused by an energy imbalance between calories taken and calories burned [3]. Thereafter, the accumulation of lipid metabolites, inflammatory signals or other factors that impede hypothalamic neuron function may also result in obesity, which may explain the biological defence of increased body fat mass [4]. Furthermore, both nature and nurture can affect an individual's likelihood of developing obesity [3]. This risk may be increased by the hereditary predisposition of one's family (the tendency to gain weight) or by a person's lifestyle choices (diet and physical activity) [5, 6].

The management of obesity comprises dietary therapy, pharmacotherapy and surgical intervention, with exercise as the cornerstone [3, 7]. Most dietary approaches focus on limiting the consumption of fat or carbohydrates to lower calorie intake [7]. According to the National Institute for Health and Care Excellence (NICE) guidelines, calorie restriction is common in successful long-term weight loss regimens, regardless of the macronutrient composition [8]. The efficacy of such regimens is contingent on diet adherence, mainly because the effects of diet on weight loss plateau with time due to compensatory adaptation [7]. For those who have not achieved a weight loss of at least 5% in six months on lifestyle changes, different pharmacological treatments can be added in patients with a BMI of ≥ 30 kg/m^2^ or a BMI of > 27 kg/m^2^ with comorbidities [7, 9, 10]. NICE guidelines have recommended only three agents for long-term weight loss: liraglutide (Saxenda), semaglutide (Wegovy) and orlistat [8]. Meanwhile, five antiobesity drugs are approved by the United States Food and Drug Administration (US-FDA): orlistat (Xenical, Alli), phentermine–topiramate (Qsymia), naltrexone–bupropion (Contrave), liraglutide (Saxenda) and semaglutide (Wegovy) [10]. The current medications for obesity tackle the underlying neurohormonal dysregulations that induce weight gain and hinder sustainable weight loss [7, 9, 10]. Bariatric surgery is the preferred option when all other therapies have failed [3, 7]. Generally, bariatric surgeries are recommended for those with a BMI of ≥ 40 kg/m^2^ or a BMI of ≥ 35 kg/m^2^ with associated comorbidities [3, 7, 8].

The two recently approved drugs, liraglutide and semaglutide, work by mimicking the activity of glucagon-like peptide 1 (GLP-1), which is a hormone that targets the hypothalamus and brainstem to regulate appetite and food intake [10]. Both medications were originally introduced as antidiabetic agents, but after showing weight loss efficacy, they were investigated as antiobesity agents. Weight loss trials focus on absolute weight reduction, alongside achieving a target weight loss of 5%, 10% and 15%, which have been proven to be clinically significant [11]. As 5%–10% weight reduction can minimise cardiovascular risk factors, ≥ 10% weight loss can improve obstructive sleep apnoea and 15% loss is associated with mortality reduction [11]. The Satiety and Clinical Adiposity—Liraglutide Evidence (SCALE) in individuals with and without diabetes trials tested the efficacy of subcutaneous 3.0 mg liraglutide once daily against placebo on overweight and obesity in diabetic and nondiabetic patients and demonstrated that liraglutide decreased body weight by 6%–8% compared to placebo's 2%–2.6% [12, 13]. Besides, they found that at Week 56, more participants in the liraglutide group achieved 5% weight loss (63%) than in the placebo group (27%) [12]. On the other hand, the Semaglutide Treatment Effect in People (STEP) with obesity trials evaluated once weekly subcutaneous 2.4-mg semaglutide versus placebo in patients with Type 2 diabetes mellitus (T2DM) and overweight or obesity [14–17]. The weight loss efficacy of semaglutide ranged between 6.2% and 12.4% from baseline. Moreover, the proportion of subjects who reached 5% weight reduction at Week 68 was higher in the semaglutide group (69%–86%) than in the placebo group (28%–48%) [15, 17]. Noticeably, both medications have been reported for gastrointestinal side effects and increasing the chances of pancreatitis [10]. Given that GLP-1 receptor agonists (RAs) are currently considered the standard pharmacological agents for obesity management, a direct comparison with tirzepatide is clinically relevant. Tirzepatide's dual action on glucose-dependent insulinotropic peptide (GIP) and GLP-1 receptors may offer additive benefits in terms of weight reduction. Establishing whether tirzepatide provides superior or comparable outcomes is important for guiding therapeutic decisions.

Tirzepatide (LY3298176) is a newly FDA-approved treatment for T2DM that shares the same pharmacological route as liraglutide and semaglutide [18]. In addition to its ability to act similarly to the GLP-1 receptor, it can also agonise the GIP receptors [18]. GIP is a hormone produced by the K cell in the intestine that stimulates glucose-dependent insulin secretion from the pancreas [18]. Theoretically, the synergetic effect of GIP and GLP-1 can produce superior antihyperglycaemic and weight loss effects [19]. The antihyperglycaemic effect has been proven by the recent SURPASS (efficacy and safety of a novel dual GIP and GLP-1 RA tirzepatide in patients with Type 2 diabetes) trials, where subcutaneous tirzepatide 5, 10 and 15 mg once weekly were compared to placebo, semaglutide, dulaglutide and basal insulin [20–25]. In a meta-analysis by Karagiannis et al., the three doses of tirzepatide lowered HbA1c more effectively than other compotators with average MD of −17.71 to −22.35 mmol/mol vs. placebo, −3.22 to −10.06 mmol/mol vs. GLP-1 RAs and −7.66 to −12.02 mmol/mol vs. basal insulin regimens [26].

The SURPASS studies showed promising effects on weight loss. Thus, the SURMOUNT (study of tirzepatide in participants with obesity or overweight) programme is currently running to evaluate tirzepatide efficacy in obesity and overweight; however, their results are expected in the next 2 years [27]. In the meantime, the SURPASS studies assessed weight loss efficacy as a secondary outcome on low sample sizes, in limited durations, and regardless of insulin use, so less is known about the extent of that efficacy. Therefore, this meta-analysis was envisaged to measure the effect of tirzepatide 5, 10 and 15 mg on obesity and overweight compared to placebo or GLP-1 RAs, despite T2DM.

## 2. Methods

We conducted a systematic review and meta-analysis to investigate the weight loss efficacy of tirzepatide in overweight/obese individuals and reported in accordance with the Preferred Reporting Items for Systematic review and Meta-Analysis (PRISMA) [28]. The study protocol was registered with the International Prospective Register of Systematic Reviews—PROSPERO (CRD-42023491599). We framed the research question based on the PICO approach:

• Population (adult with overweight or obesity)
• Intervention (once weekly subcutaneous tirzepatide 5, 10 and 15 mg)
• Control (placebo or GLP-1 RAs)
• Outcome (absolute change in weight from baseline)

### 2.1. Search Strategy

We searched PubMed, Embase and the Cochrane Central Register of Controlled Trials (CENTRAL) databases from their inception up until 31st July 2023 for clinical trials that compared once weekly tirzepatide (5, 10 and 15 mg) with placebo or GLP-1 RAs in adults with overweight or obesity. The terms “tirzepatide” OR “LY3298176” AND “weight loss” AND “obesity” were used to search the databases mentioned above. The search was kept broad to capture all related studies as the medication is new, and the intended outcome is often reported secondarily. Further information was retrieved from the American Diabetes Association (ADA), Clinicaltrials.gov and clinicaltrials.eu websites.

### 2.2. Eligibility Criteria

We included studies that fulfiled the following inclusion criteria: randomised clinical trials that compared the intended agents and measured weight loss for a duration of at least 20 weeks. Eligible participants were adults who were obese or overweight, regardless of the use of oral hypoglycaemic agents (OHAs), insulin or the presence of diabetes. We restricted our search to the English language only. We excluded studies with a duration of less than 20 weeks, those without a comparator or those who used insulin as a comparator. We selected a minimum study duration of 20 weeks to ensure that weight loss outcomes reflected sustained pharmacological effects rather than transient changes. This threshold is consistent with prior meta-analyses evaluating antiobesity treatments and increases the validity and comparability of efficacy and safety outcomes across studies.

### 2.3. Selection Process

Two authors (AK, AAJ) independently filtered the matching studies for duplication and screened at the title and abstract level; thereafter, eligible studies were examined manually through a schedule in which the name of the study, interventions, duration and outcomes were reported. Then, the schedule was filtered for meeting an exclusion criterion. Any discrepancies were resolved by consensus in consultation with a third reviewer (RR).

### 2.4. Data Collection

Characteristics and intended outcomes of included trials were added to a self-designed extraction form in Microsoft Excel (Microsoft Corporation, WA, USA). The primary outcome focused on the efficacy of tirzepatide (5, 10 and 15 mg) in weight loss from baseline. Secondary outcomes involved achievement of ≥ 5%, 10% and 15% body weight reduction from baseline and the safety profile of tirzepatide in terms of gastrointestinal side effects (nausea, vomiting, diarrhoea and constipation) and serious adverse events (AEs). Mean, standard deviation and the number of participants were recorded for continuous outcomes. Conversely, dichotomous outcomes were recorded based on the number of participants who experienced at least one event of interest. Data extraction was based on the ‘modified intention to treat' for both outcomes. In case of any missing data, primary investigators or publications' sites were contacted for further information. We used the Cochrane risk-of-bias tool for randomised controlled trials (RCTs) (Version 2) to assess the risk of bias in each study, and the risk was considered low if the study's evaluation did not involve any high risk in all domains [29].

### 2.5. Data Analysis

We performed the statistical analysis using Review Manager (RevMan) Version 5.4.1. For the continuous outcome, mean differences (MDs) and 95% CIs were calculated using an inverse variance random-effects model. For dichotomous outcomes, odds ratio or risk ratio along with 95% CIs were calculated using the Mantel–Haenszel method. A *p* value of < 0.05 was considered statistically significant. For both comparisons, a post hoc sensitivity analysis was performed, excluding one trial with the shortest duration and one trial with the longest duration. A more granular sensitivity analysis was not performed, as the included studies demonstrated consistent effect estimates with overlapping confidence intervals and no substantial influence of individual studies on the pooled outcomes. Statistical heterogeneity was calculated by *I*^2^ statistic, where an *I*^2^ value of more than 75% indicated a high heterogeneity. If the *I*^2^ value was found to be above 50% then a random-effects model was chosen or a fixed effect model was the choice for analysing the outcomes. Publication bias was accounted for through a funnel plot and an Egger's test, which was performed on Jamovi 2.2.5. The GRADE profiler (GRADEpro GDT) was then used to assess evidence quality for each review outcome. Using GRADE, evidence quality is downgraded from ‘high quality' by one level for each serious issue identified in the domains of risk of bias, imprecision, indirectness, inconsistency and publication bias [30].

## 3. Results

### 3.1. Study Selection

**Figure 1:**
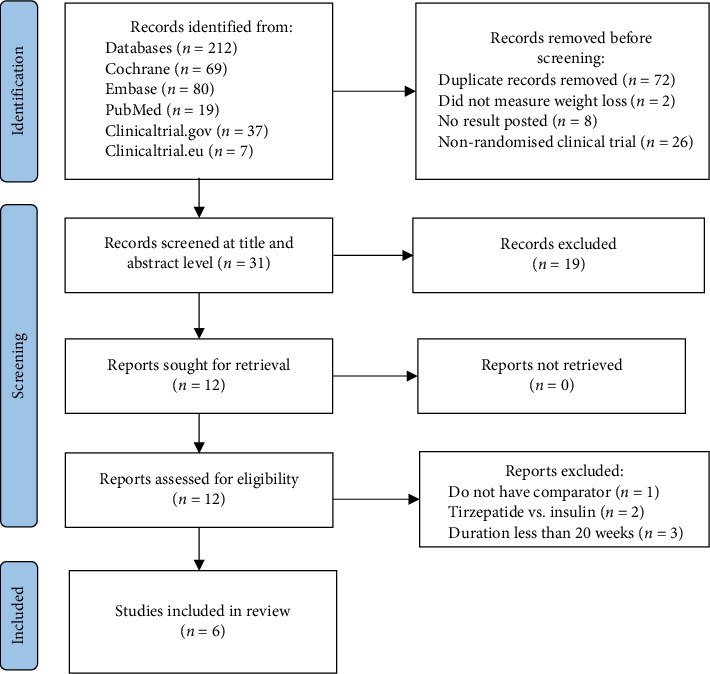
The PRISMA flowchart for RCT screening.

Our primary search from databases yielded 212 studies, of which six RCTs [20, 23–25, 31, 32] met the inclusion criteria and were included in this meta-analysis. The data of 6266 participants were evaluated for the primary and secondary outcomes. Moreover, three studies appeared to share most of the inclusion criteria, yet they were excluded for two reasons [21, 22, 33]. SURPASS-3 and SURPASS-4 compared tirzepatide with different types of insulin, which would be beneficial in measuring the antihyperglycaemic effect but not weight loss; thus, they were excluded [21, 33]. SURPASS J-combo compared different doses of tirzepatide in diabetic patients on OHAs, so there was no comparator; therefore, this study was also excluded [22]. The process of inclusion of articles is summarised in the flowchart in Figure 1.

### 3.2. Study Characteristics

All studies were multicentres and carried out in a double-blind approach, except for one study (SURPASS-2), which was an open-label study (studies' details are in Table 1). Participants were mostly diabetic with overweight or obese, defined as a BMI of ≥ 23 kg/m^2^ in four studies (SURPASS-1, Juan P. Frias et al., SURPASS-5, and SURPASS J-mono) and a BMI of ≥ 25 kg/m^2^ in one study (SURPASS-2). In the sixth study, Jastreboff et al. included nondiabetic participants with a BMI of ≥ 30 or ≥ 27 kg/m^2^ with comorbidities [32]. Generally, the control group consisted of a placebo in four trials and GLP-1 RAs (semaglutide 1 mg or dulaglutide 1.5 and 0.75 mg) in three trials. Juan P. Frias et al. involved both controls in their study [31]. Overall, the six studies tested different doses of tirzepatide (5, 10 and 15 mg) with a dose escalation of 2.5 mg over 4 weeks. Nevertheless, Juan P. Frias et al. followed a different dose-escalation of 5 mg every 2 weeks [31]. The sample size ranged between 264 and 2539 with a duration of 26–72 weeks.

### 3.3. Risk of Bias

**Figure 2:**
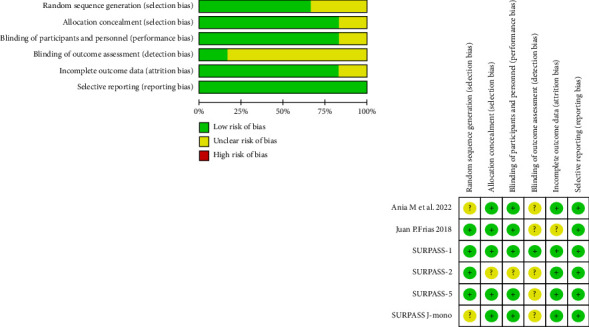
The Cochrane risk-of-bias graph and diagram.

After performing the Cochrane risk-of-bias tool, included studies were considered high quality trials with a low risk of bias (Figure 2).

### 3.4. Efficacy Outcomes

**Figure 3:**
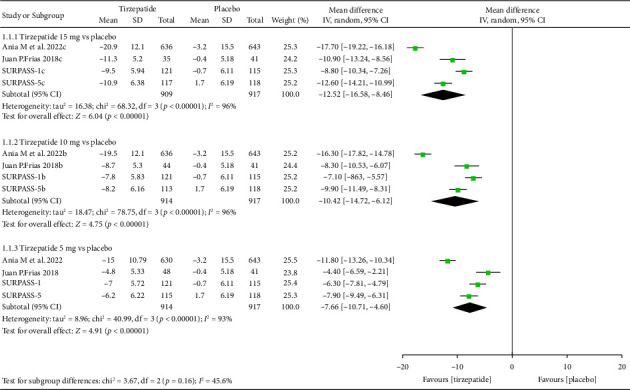
Absolute change in body weight of tirzepatide (5, 10 and 15 mg) compared to placebo.

Approximately 910 participants in each group received either tirzepatide (5, 10 or 15 mg) or placebo (Figure 3). Tirzepatide 5, 10 and 15 mg decreased weight compared to placebo with a MD of −7.66 kg (95% CI, −10.71 to −4.60), −10.42 kg (95% CI, −14.72 to −6.12) and −12.52 kg [9(5% CI, −16.58 to −8.46), respectively, and *p* value of less than 0.001 for all tests (Figure 3). In a sensitivity analysis that excluded a trial with the shortest and longest durations, statistical heterogeneity was reduced, and the results were consistent.

Moreover, the percentages of participants who achieved 5% weight reduction were higher in the tirzepatide groups (76%, 80% and 86% for tirzepatide 5, 10 and 15 mg, respectively) in comparison to 26% in the placebo arm. Similarly, the proportion of those who accomplished 10% and 15% weight loss was higher in the tirzepatide groups against the placebo (Table 2).

**Figure 4:**
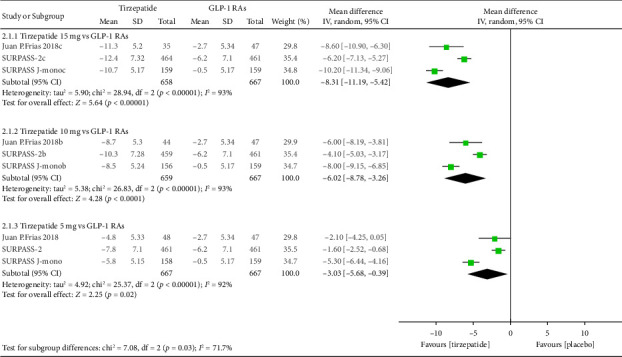
Absolute change in body weight of tirzepatide (5, 10 and 15 mg) compared to GLP-1 receptor agonists.

In comparing tirzepatide and GLP-1 RAs, the data of at least 658 participants in each group were analysed for the MD of body weight from baseline (Figure 4). The three different doses of tirzepatide (5, 10 and 15 mg) showed greater efficacy than GLP-1 RAs (−3.03 kg [95% CI, −5.68 to −0.39], −6.02 kg [95% CI, −8.78 to −3.26] and −8.31 kg [95% CI, −11.19 to −5.42], respectively) with *p* value of < 0.02 for all (Figure 4). In a sensitivity analysis that excluded the shortest and longest duration trials, statistical heterogeneity was reduced, and the results were consistent.

Furthermore, the percentage of participants who reached a target weight loss of 5% was 64% with tirzepatide 5 mg, 80% with tirzepatide 10 mg and 84% with tirzepatide 15 mg compared to 44% with GLP-1 RAs. Also, the three doses of tirzepatide appeared to have higher percentages of subjects achieving 10% and 15% weight reduction than GLP-1 RAs (Table 2).

### 3.5. Safety Outcomes

The risk of gastrointestinal side effects was higher in all tirzepatide doses than in placebo, especially with the 15-mg dose (diarrhoea: RR, 2.89, 95% CI [2.24, 3.74], nausea: RR, 3.55, 95% CI [2.79, 4.51] and vomiting: RR, 6.68, 95% CI [4.05, 11.04]) (Table 3). However, constipation occurred more with the 10-mg dose (RR, 3.22, 95% CI [2.30, 4.53]) (Table 3). On the other hand, tirzepatide doses and GLP-1 RAs appeared identical in the risk of causing GIT side effects. Nevertheless, diarrhoea happened slightly more in tirzepatide 10 mg and 15 mg than in GLP-1 RAs (RR, 1.40, 95% CI [1.06, 1.85]) and (RR, 1.35, 95% CI [1.02, 1.79]), respectively, and the risk of developing nausea was higher in tirzepatide 15 mg than in GLP-1 RAs (RR, 1.40, 95% CI [1.13, 1.74]) (Table 3). Regarding serious side effects, all tirzepatide doses did not statistically differ from GLP-1 RAs or placebo as the risk ratios ranged between 0.75 and 1.28 with 95% CI, respectively (Table 3).

### 3.6. Publication Bias

**Figure 5:**
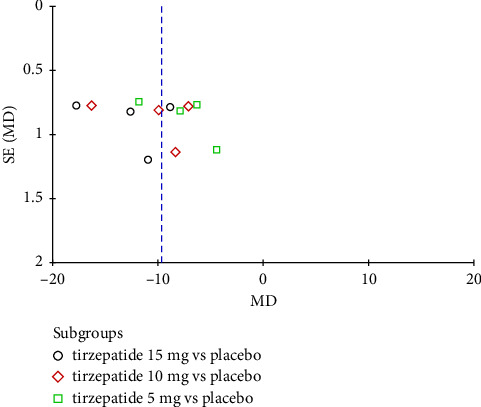
Funnel plot of tirzepatide vs. placebo.

**Figure 6:**
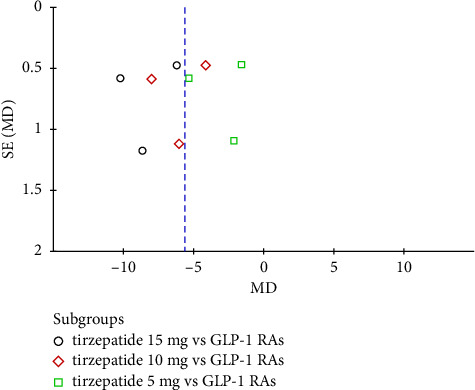
Funnel plot of tirzepatide vs. GLP-1 receptor agonists.

Although the funnel plots of tirzepatide vs. placebo (Figure 5) and tirzepatide vs. GLP-1 RA (Figure 6) appeared symmetrical, the review included less than 10 studies, and it is unpractical to visually interpret the funnel plot to detect publication bias [34]. Thus, Egger's test was calculated for both comparisons, demonstrating no publication bias as the *p* value was higher than 0.05 (*p*=0.278 and *p*=0.843, respectively).

### 3.7. GRADE Assessment

As part of the evidence quality appraisal, a GRADE assessment was performed for the primary outcome of weight loss. The evidence for achieving ≥ 15% weight reduction with tirzepatide (5, 10 and 15 mg) compared to placebo was rated as high certainty, with odds ratios ranging from 9.85 to 26.03 and consistent findings across trials (Supporting Table 10). In contrast, evidence for ≥ 10% and ≥ 5% weight loss was rated as low certainty due to serious inconsistency and suspected publication bias, despite strong associations. For absolute weight change from baseline, the 15-mg and 10-mg doses showed moderate certainty while the 5-mg dose was supported by low certainty evidence. These ratings reflect both the magnitude of effect and limitations in consistency and reporting across included studies.

## 4. Discussion

In this meta-analysis, we combined and analysed the data of six updated RCTs that looked into the efficacy of tirzepatide (5, 10 and 15 mg) once weekly against placebo or GLP-1 RAs in adults with obesity or overweight. Our findings showed that tirzepatide provided a more significant dose-dependent weight loss effect than placebo and the GLP-1 RAs, dulaglutide and semaglutide. The effect remained significant even after a post hoc analysis in which trials with the longest and shortest duration were eliminated. Moreover, the clinically significant 5%, 10% and 15% weight loss from baseline happened more in the tirzepatide groups than in the other groups. The safety outcomes revealed that tirzepatide and GLP-1 RAs were similar in terms of the odds of gastrointestinal side effects and serious side effects. However, when compared to placebo, tirzepatide groups had more incidence of GIT side effects, yet they appeared to be similar in the odds of serious side effects.

In our literature search, we identified two previous reviews that focused on the hypoglycaemic effects of tirzepatide and assessed weight loss as a secondary outcome [26, 35]. Bhagavathula et al. pooled the data of GLP-1 RA and placebo together and compared them to tirzepatide. This can make interpreting the result difficult as, unlike placebo, GLP-1 RAs have a well-established effect on weight loss. In the other review, Karagiannis et al. included only those with T2DM since their primary outcome was the hypoglycaemic effect. Despite the presence of these cofactors in both reviews, tirzepatide showed a significant weight loss efficacy with the MD ranging from −6.31 to −9.36 kg compared to placebo and −1.68 to −7.16 compared to GLP-1 RAs in Karagiannis et al.'s study and −7.0 to −8.6 kg in Bhagavathula et al.'s study. Our analysis differs from these two reviews by including subjects with obesity or overweight despite T2DM and adding two recent studies, one of which evaluated weight loss as a primary outcome [23, 32]. Additionally, our findings point towards a more potent effect of tirzepatide than these analyses, with the MD averaging between −7.66 and −12.52 against placebo and −3.03 and −8.31 against GLP-1 RAs.

Our study results were in concordance with the Mesquita et al.'s study, which compared tirzepatide with a placebo for the coprimary endpoints of absolute and percent change in weight [36]. Results showed that tirzepatide at 5-, 10- and 15-mg doses was more effective than the placebo, resulting in a significant decrease in body weight (−7.7 kg [95% CI −11.0, −4.4; *p* < 0.001], −11.6 kg [95% CI −18.8, −4.3; *p*=0.002], and −11.8 kg [95% CI −17.4, −6.2; *p* < 0.001], respectively) and a decrease in percent weight change. However, our study demonstrated that constipation occurred more with the 10-mg dose (OR, 3.57 [95% CI, 2.47–5.18]).

Compared to the data of the two similar agents, liraglutide and semaglutide, tirzepatide showed a more promising weight loss effect. In a meta-analysis by Moon et al., 31 studies (8060 participants) were included in their review, and they found that liraglutide (1.8 mg and 3 mg daily) decreased weight by 4.19 kg (95% CI, −4.84 to −3.55) compared to placebo [37].

Konwar et al. conducted another meta-analysis which demonstrated that liraglutide 3.0 mg daily decreased body weight (mean reduction of 4.9 kg [95% CI = −5.43, −4.39]), waist circumference (mean reduction of 3.5 cm [95% CI = −4.21, −2.89]), and BMI (mean reduction of 1.86 kg/m^2^ [95% CI = −2.14, −1.57]) from baseline when compared with placebo [38]. On the other hand, Zhong et al. analysed four RCTs that measured the effect of semaglutide 2.4 mg once weekly vs. placebo on weight loss [39]. The result revealed that semaglutide minimised weight by −11.90 kg (95% CI, −13.24 to −10.56). In this review, we found that once-weekly tirzepatide (5, 10 and 15 mg) was able to decrease weight in comparison to placebo by −7.66 kg (95% CI, −10.71 to −4.60, −10.42 kg (95% CI, −14.72 to −6.12) and −12.52 kg (95% CI, −16.58 to −8.46), respectively. Furthermore, the percentage of participants who accomplished a target weight loss of 5% was only 63% with liraglutide at 56 weeks in the SCALE obesity and prediabetes trial and 69% with semaglutide at 68 weeks in the STEP-2 study, whereas it was 85% with 5-mg tirzepatide, 88% with 10-mg tirzepatide, and 90% with 15-mg tirzepatide at 72 weeks in the SURMOUNT-1 trial [12, 17, 32].

Our study results were in accordance with the meta-analysis conducted by Tan et al., which found that tirzepatide was more effective in weight loss than placebo and semaglutide [40]. A more significant reduction was observed with higher doses, and a dose-dependent increase was observed with 10 and 15 mg. Our study compared the efficacy and safety of tirzepatide and placebo and other GLP-1s (both semaglutide 1 mg, dulaglutide 0.75/1.5 mg); however, Tan et al. have included studies with placebo, insulin and semaglutide as a comparator (not included studies with dulaglutide). Nonetheless, the dose of tirzepatide, ranging from 5 to 15 mg, significantly impacted weight loss (≥ 5%) compared to placebo in 65.91%, 80.72% and 86.95% of patients, respectively (as against 76%, 80% and 86% for tirzepatide 5, 10 and 15 mg, respectively, in our study). The study also found no significant differences in gastrointestinal AEs between tirzepatide and semaglutide, except for diarrhoea with tirzepatide 10 mg (OR 1.51, 95% CI: 1.04–2.19). However, our study results demonstrated that diarrhoea happened slightly more in the tirzepatide 10 and 15 mg than in GLP-1 RAs (OR, 1.48 [95% CI, 1.07–2.04] and OR, 3.09 [95% CI, 1.74–5.48], respectively), and the odds of nausea was higher in the tirzepatide 15 mg than in GLP-1 RAs (OR, 1.73 [95% CI, 1.03–2.92).

Lin et al. evaluated the safety and efficacy of three doses of tirzepatide compared to placebo, GLP-1 RAs and insulin in adult patients with or without diabetes [41]. The study demonstrated that patients' body weight was significantly reduced by 1.05 kg (95% CI (−1.48, −0.63) when compared with GLP-1 RAs (semaglutide/dulaglutide group) as against our study 3.03 kg (95% CI (−5.68, −0.39). The meta-analysis revealed a significant difference in the incidence of gastrointestinal side effects between the tirzepatide and placebo groups, which is in accordance with our study. However, there was no significant difference in the incidence of AEs in the tirzepatide 5 mg (OR = 1.01, 95% CI [0.80, 1.28], *p*=0.92) and 10 mg (OR = 1.17, 95% CI [0.92, 1.48], *p*=0.2) groups when compared to GLP-1 RAs. Our study also demonstrated that tirzepatide doses and GLP-1 RAs appeared identical in the **risk** of causing gastrointestinal side effects. Nevertheless, the risk of diarrhoea was slightly higher with tirzepatide 10 mg (RR 1.40, 95% CI [1.06, 1.85]) and 15 mg (RR 1.35, 95% CI [1.02, 1.79]) when compared to GLP-1 RAs. Similarly, the risk of developing nausea was greater with tirzepatide 15 mg (RR 1.40, 95% CI [1.13, 1.74]) than GLP-1 RAs. These findings suggest a dose-related trend in gastrointestinal intolerance. Furthermore, the incidence of serious AEs did not significantly differ between tirzepatide and GLP-1 RAs or placebo, with risk ratios ranging between 0.75 and 1.28 across comparisons. These safety findings are in line with those reported by Lin et al., who observed no major differences in serious AEs between treatment groups and support the overall tolerability of tirzepatide across doses.

Although several included trials also reported glycaemic outcomes such as HbA1c or fasting plasma glucose, these parameters were not uniformly available or reported across studies in a manner suitable for pooled analysis. Moreover, they were often reported as primary outcomes in diabetic cohorts. As our focus was on weight loss efficacy in adults with obesity or overweight, we did not include metabolic endpoints. Future meta-analyses should aim to integrate both glycaemic and weight-related outcomes to provide a more holistic assessment of tirzepatide's therapeutic potential.

The findings of this review must be interpreted carefully as the *I*^2^ statistic indicated a high degree of heterogeneity, especially in the primary outcome. This could be attributed to the variation in patient's baseline characteristics, particularly OHA or insulin use, sample sizes and studies' durations. Even though Egger's test was not statistically significant, publication bias cannot be rolled out since positive studies are more likely to be published than negative ones, particularly if they were sponsored by the medication's manufacturer, which is the case for included studies. The data in this report were retrieved based on study-level data, yet individual participant data would produce more statically and clinically robust results. Moreover, the included studies compared tirzepatide doses versus lower doses of GLP-1 RAs (dulaglutide 0.75 mg, 1.5 mg and semaglutide 1 mg). Thus, it is difficult to estimate the extent of tirzepatide weight loss efficacy against GLP-1 RAs.

Additionally, subgroup analyses based on diabetes status or baseline BMI could not be conducted, as only one trial included nondiabetic participants and BMI-stratified outcomes were not reported in the included studies. Future trials should aim to report outcomes by these characteristics to better understand if the efficacy and safety of tirzepatide vary across metabolic phenotypes or degrees of adiposity. Furthermore, our safety analysis was limited to gastrointestinal AEs and serious AEs, as other off-target effects such as hypoglycaemia, pancreatitis or cardiovascular events were either not uniformly reported or not quantified across the included studies. Future meta-analyses would benefit from a broader safety evaluation as more detailed safety data become available. Another important limitation is the lack of data on body composition changes across the included trials. Although tirzepatide showed robust weight loss efficacy, the recent evidence from a network meta-analysis has indicated that it may be associated with a greater reduction in lean mass than other GLP-1 RAs. This is a clinically significant concern, as excessive lean mass loss can negatively impact metabolic health and physical function [42]. Future trials should incorporate assessments of body composition to better characterise the qualitative aspects of weight loss.

This review could potentially assist healthcare providers and policymakers in deciding whether tirzepatide use is advantageous for obesity or overweight. Although head-to-head trials between tirzepatide and higher doses of GLP-1 RAs are still expected, these findings suggest that tirzepatide is reasonably favourable to liraglutide and semaglutide in terms of weight loss efficacy and comparable to the safety profiles of those agents. Nonetheless, it is currently unknown whether tirzepatide can produce cardiovascular advantages comparable to those of specific GLP-1 RAs or sodium–glucose Cotransporter 2 inhibitors [43]. Notably, the SURPASS-CVOT trial is anticipated to produce firm answers to the cardioprotectiveness of tirzepatide as it compares with dulaglutide on MACE in participants with T2DM. Future studies must focus on directly comparing tirzepatide with semaglutide or liraglutide in regards of their effect on lipid profile, metabolic comorbidities, adherence and cost-effectiveness.

## 5. Conclusion

Our meta-analysis evaluated the efficacy of subcutaneous tirzepatide (5 mg, 10 mg and 15 mg once weekly) with placebo or GLP-1 RAs in people with obesity or overweight. Compared to placebo and GLP-1 RAs, dulaglutide and semaglutide, tirzepatide produced a larger dose-dependent effect on weight loss. Additionally, the clinically relevant 5%, 10% and 15% weight reduction from baseline occurred more frequently in the tirzepatide groups than in the other groups. Regarding the likelihood of gastrointestinal side effects and serious AEs, tirzepatide and GLP-1 RAs demonstrated comparable safety profiles. However, this efficacy must be balanced against potential drawbacks such as gastrointestinal side effects, reduced tolerability at higher doses and emerging concerns regarding lean mass loss. Future studies should prioritise a more holistic evaluation of weight loss interventions by including functional and metabolic outcomes beyond absolute weight reduction.

## Figures and Tables

**Table 1:** Characteristics of included studies.

Study	Duration (weeks)	Blinding status	Oral hypoglycaemic agents or insulin	Intervention vs. control	Participants	Mean body weight (kg)	Change in body weight (kg)
Jastreboff et al. 2022 [32]	72	Double-blind	Nondiabetic patients	Tirzepatide 5 mg	630	102.9	−15.7
				Tirzepatide 10 mg	636	105.8	−20.4
				Tirzepatide 15 mg	630	105.6	−21.9
				Placebo	643	104.8	−3.2

SURPASS-2 [25]	40	Open label	Diabetic patients on metformin at least 1500 mg.	Tirzepatide 5 mg	470	92.5	−7.8
				Tirzepatide 10 mg	469	94.8	−10.3
				Tirzepatide 15 mg	470	93.8	−12.4
				Semaglutide 1 mg	469	93.7	−6.2

SURPASS-1 [20]	40	Double-blind	Diabetic patients (drug naïve or on previous OHAs)	Tirzepatide 5 mg	121	87	−7.0
				Tirzepatide 10 mg	121	86.2	−7.8
				Tirzepatide 15 mg	121	85.4	−9.5
				Placebo	115	84.8	−0.7

SURPASS J-mono [23]	52	Double-blind	Diabetic patients (drug naïve or on previous OHAs)	Tirzepatide 5 mg	158	78.5	−5.8
				Tirzepatide 10 mg	156	78.9	−8.5
				Tirzepatide 15 mg	159	78.9	−10.7
				Dulaglutide 0.75 mg	159	76.5	−0.5

SURPASS-5 [24]	40	Double-blind	Diabetic patients on insulin glargine ± metformin	Tirzepatide 5 mg	116	95.5	−6.2
				Tirzepatide 10 mg	119	95.4	−8.2
				Tirzepatide 15 mg	120	96.2	−10.9
				Placebo	120	94.1	+1.7

Frias et al. 2018 [31]	26	Double-blind	Diabetic patients (drug naïve or on metformin)	Tirzepatide 5 mg	55	92.8	−4.8
				Tirzepatide 10 mg	51	92.7	−8.7
				Tirzepatide 15 mg	53	89.1	−11.3
				Dulaglutide 1.5 mg	54	89.8	−2.7
				Placebo	51	91.5	−0.4

**Table 2:** Participants achieving bodyweight targets of (≥ 5%, ≥ 10% and ≥ 15%).

Intervention	Control	Participants with outcome/total number of participants and percentage of participants achieving the outcome		OR (95% CI)	*I* ^2^ (%)
		Intervention	Control		
*≥ 5% weight reduction*					
Tirzepatide 5 mg	Placebo	705/921 (76%)	245/925 (26%)	12.29 (8.83–17.12)	17
	GLP-1 RAs	438/683 (64%)	299/681 (44%)	3.89 (0.93–16.37)	95

Tirzepatide 10 mg	Placebo	766/921 (80%)	245/925 (26%)	20.19 (12.19–33.42)	48
	GLP-1 RAs	542/676 (80%)	299/681 (44%)	9.79 (1.82–52.55)	96

Tirzepatide 15 mg	Placebo	794/920 (86%)	245/925 (26%)	31.59 (14.34–69.58)	75
	GLP-1 RAs	575/681 (84%)	299/681 (44%)	11.84 (2.05–68.41)	96

*≥ 10% weight reduction*					
Tirzepatide 5 mg	Placebo	501/922 (54%)	123/927 (13%)	15.45 (6.31–37.84)	33
	GLP-1 RAs	223/683 (32%)	123/682 (18%)	3.59 (0.82–15.70)	91

Tirzepatide 10 mg	Placebo	612/927 (66%)	123/927 (13%)	35.66 (10.38–117.45)	55
	GLP-1 RAs	318/676 (47%)	123/682 (13%)	7.83 (1.67–36.78)	92

Tirzepatide 15 mg	Placebo	649/923 (70%)	123/927 (13%)	36.94 (14.83–91.97)	34
	GLP-1 RAs	395/682 (58%)	123/682 (13%)	11.23 (2.10–60.09)	93

*≥ 15% weight reduction*					
Tirzepatide 5 mg	Placebo	329/922 (36%)	56/927 (6%)	9.85 (7.22–13.42)	0
	GLP-1 RAs	99/683 (14%)	40/682 (6%)	4.95 (0.82–29.96)	75

Tirzepatide 10 mg	Placebo	482/927 (52%)	56/927 (6%)	21.45 (15.67–29.35)	0
	GLP-1 RAs	163/676 (24%)	40/682 (6%)	11.76 (1.78–77.84)	79

Tirzepatide 15 mg	Placebo	516/923 (56%)	56/927 (6%)	26.03 (18.95–35.74)	0
	GLP-1 RAs	253/682 (37%)	40/682 (6%)	20.66 (2.80–152.59)	81

**Table 3:** Adverse events with different doses of tirzepatide compared to GLP-1 receptor agonists or placebo.

Intervention	Control	Events/number of participants		RR (95% CI)	*I* ^2^ (%)
		Intervention	Control		
*Constipation*					
Tirzepatide 5 mg	Placebo	122/922	40/929	3.08 [2.19, 4.33]	0
	GLP-1 RAs	59/684	45/682	1.31 [0.90, 1.90]	0

Tirzepatide 10 mg	Placebo	129/927	40/929	3.22 [2.30, 4.53]	0
	GLP-1 RAs	55/678	47/682	1.18 [0.81, 1.71]	0

Tirzepatide 15 mg	Placebo	92/924	40/929	2.32 [1.62, 3.31]	0
	GLP-1 RAs	45/683	47/682	0.95 [0.65, 1.41]	0

*Diarrhoea*					
Tirzepatide 5 mg	Placebo	159/922	70/929	2.70 [2.05, 3.56]	49
	GLP-1 RAs	102/684	74/682	1.37 [1.04, 1.82]	51

Tirzepatide 10 mg	Placebo	164/927	61/929	2.70 [2.05, 3.56]	49
	GLP-1 RAs	103/678	74/682	1.40 [1.06, 1.85]	0

Tirzepatide 15 mg	Placebo	201/924	70/929	2.89 [2.24, 3.74]	51
	GLP-1 RAs	100/683	74/682	1.35 [1.02, 1.79]	0

*Nausea*					
Tirzepatide 5 mg	Placebo	195/922	74/929	2.66 (2.07, 3.42)	0
	GLP-1 RAs	112/684	112/682	1.00 [0.79, 1.26]	35

Tirzepatide 10 mg	Placebo	260/927	74/929	3.53 [2.78, 4.49]	0
	GLP-1 RAs	132/678	112/682	1.19 [0.95, 1.49]	77

Tirzepatide 15 mg	Placebo	260/924	74/929	3.55 [2.79, 4.51]	7
	GLP-1 RAs	157/683	112/682	1.40 [1.13, 1.74]	59

*Vomiting*					
Tirzepatide 5 mg	Placebo	68/922	17/929	4.04 [2.40, 6.82]	0
	GLP-1 RAs	45/683	45/683	0.95 [0.64, 1.42]	76

Tirzepatide 10 mg	Placebo	88/927	18/929	4.91 [2.98, 8.07]	45
	GLP-1 RAs	56/678	46/682	1.23 [0.84, 1.78]	40

Tirzepatide 15 mg	Placebo	113/924	17/929	6.68 [4.05, 11.04]	0
	GLP-1 RAs	79/683	46/682	1.72 [1.21, 2.43]	80

*Serious side effects*					
Tirzepatide 5 mg	Placebo	55/922	59/929	0.95 [0.66, 1.35]	0
	GLP-1 RAs	42/684	30/682	1.20 [0.75, 1.92]	71

Tirzepatide 10 mg	Placebo	62/927	59/929	1.06 [0.75, 1.49]	0
	GLP-1 RAs	38/678	30/682	1.28 [0.80, 2.03]	45

Tirzepatide 15 mg	Placebo	44/924	59/929	0.75 [0.52, 1.10]	0
	GLP-1 RAs	36/683	30/682	1.20 [0.75, 1.92]	87

## Data Availability

The datasets generated during the current study are available from the corresponding author upon reasonable request.

## Supporting Information

Supporting Table 1 depicts the quality of evidence using the GRADE assessment for the primary outcome of weight loss with three doses of tirzepatide vs GLP-1 agonists or placebo. The evidence for achieving ≥ 15% weight reduction with tirzepatide (5, 10, and 15 mg) compared to placebo was rated as high certainty, with odds ratios ranging from 9.85 to 26.03 and consistent findings across trials.
