# Supplementary material for: Weight Loss Efficacy of Tirzepatide Compared to Placebo or GLP-1 Receptor Agonists in Adults With Obesity or Overweight: A Meta-Analysis of Randomized Controlled Trials With ≥ 20 Weeks Treatment Duration
Source: J Obes. 2025 Jul 24;2025:3442754. doi: 10.1155/jobe/3442754 (PMC12313391; doi:10.1155/jobe/3442754)
Supplement: Supporting Information — Additional supporting information can be found online in the Supporting Information section. [file 3442754.f1.docx]

**Supplementary Table 1: Quality of evidence using GRADE criteria**

**Question:** Tirzepatide compared to GLP-1 or placebo for weight loss

| **Certainty assessment** | | | | | | | **№ of patients** | | **Effect** | | **Certainty** | **Importance** |
| --- | --- | --- | --- | --- | --- | --- | --- | --- | --- | --- | --- | --- |
| **№ of studies** | **Study design** | **Risk of bias** | **Inconsistency** | **Indirectness** | **Imprecision** | **Other considerations** | **tirzepatide** | **placebo** | **Relative (95% CI)** | **Absolute (95% CI)** |  |  |
| **Absolute change in weight from baseline (kg) - tirzepatide 15mg vs placebo** | | | | | | | | | | | | |
| 4 | randomised trials | not serious | very serious | not serious | not serious | publication bias strongly suspected very strong association | 909 | 917 | - | MD **12.52 lower** (16.58 lower to 8.46 lower) | ⨁⨁⨁◯ Moderate |  |
| **Absolute change in weight from baseline (kg) - tirzepatide 10mg vs placebo** | | | | | | | | | | | | |
| 4 | randomised trials | not serious | very serious | not serious | not serious | publication bias strongly suspected very strong association | 914 | 917 | - | MD **10.42 lower** (14.72 lower to 6.12 lower) | ⨁⨁⨁◯ Moderate |  |
| **Absolute change in weight from baseline (kg) - tirzepatide 5mg vs placebo** | | | | | | | | | | | | |
| 4 | randomised trials | not serious | very serious | not serious | not serious | publication bias strongly suspected strong association | 914 | 917 | - | MD **7.66 lower** (10.71 lower to 4.6 lower) | ⨁⨁◯◯ Low |  |
| **> or = 5% body weight reduction - tirzepatide 5mg vs placebo** | | | | | | | | | | | | |
| 4 | randomised trials | not serious | very serious | not serious | not serious | publication bias strongly suspected strong association | 705/921 (76.5%) | 245/925 (26.5%) | **OR 12.29** (8.83 to 17.12) | **551 more per 1,000** (from 496 more to 596 more) | ⨁⨁◯◯ Low |  |
| **> or = 5% body weight reduction - tirzepatide 10mg vs placebo** | | | | | | | | | | | | |
| 4 | randomised trials | not serious | very serious | not serious | not serious | publication bias strongly suspected strong association | 766/921 (83.2%) | 245/925 (26.5%) | **OR 20.19** (12.19 to 33.42) | **614 more per 1,000** (from 550 more to 658 more) | ⨁⨁◯◯ Low |  |
| **> or = 5% body weight reduction - tirzepatide 15mg vs placebo** | | | | | | | | | | | | |
| 4 | randomised trials | not serious | very serious | not serious | not serious | publication bias strongly suspected strong association | 794/920 (86.3%) | 245/925 (26.5%) | **OR 31.59** (14.34 to 69.58) | **654 more per 1,000** (from 573 more to 697 more) | ⨁⨁◯◯ Low |  |
| **10% body weight - 5mg vs placebo** | | | | | | | | | | | | |
| 4 | randomised trials | not serious | very serious | not serious | not serious | publication bias strongly suspected strong association | 501/922 (54.3%) | 123/927 (13.3%) | **OR 15.45** (6.31 to 37.84) | **570 more per 1,000** (from 358 more to 720 more) | ⨁⨁◯◯ Low |  |
| **10% body weight - 10mg vs placebo** | | | | | | | | | | | | |
| 4 | randomised trials | not serious | very serious | not serious | not serious | publication bias strongly suspected strong association | 612/927 (66.0%) | 123/927 (13.3%) | **OR 35.66** (10.83 to 117.45) | **712 more per 1,000** (from 491 more to 815 more) | ⨁⨁◯◯ Low |  |
| **10% body weight - 15mg vs placebo** | | | | | | | | | | | | |
| 4 | randomised trials | not serious | not serious | not serious | not serious | publication bias strongly suspected strong association | 649/923 (70.3%) | 123/927 (13.3%) | **OR 36.94** (14.83 to 91.97) | **717 more per 1,000** (from 561 more to 801 more) | ⨁⨁⨁⨁ High |  |
| **15% body weight loss - 5mg vs placebo** | | | | | | | | | | | | |
| 4 | randomised trials | not serious | not serious | not serious | not serious | publication bias strongly suspected strong association | 329/922 (35.7%) | 56/927 (6.0%) | **OR 9.85** (7.22 to 13.42) | **327 more per 1,000** (from 257 more to 403 more) | ⨁⨁⨁⨁ High |  |
| **15% body weight loss - 10mg vs placebo** | | | | | | | | | | | | |
| 4 | randomised trials | not serious | not serious | not serious | not serious | publication bias strongly suspected strong association | 482/927 (52.0%) | 56/927 (6.0%) | **OR 21.45** (15.67 to 29.35) | **519 more per 1,000** (from 441 more to 593 more) | ⨁⨁⨁⨁ High |  |
| **15% body weight loss - 15mg vs placebo** | | | | | | | | | | | | |
| 4 | randomised trials | not serious | not serious | not serious | not serious | publication bias strongly suspected strong association | 516/923 (55.9%) | 56/927 (6.0%) | **OR 26.03** (18.95 to 35.74) | **566 more per 1,000** (from 489 more to 636 more) | ⨁⨁⨁⨁ High |  |

**CI:** confidence interval; **MD:** mean difference; **OR:** odds ratio

GRADE Working Group grades of evidence: High quality, further research is very unlikely to change our confidence in the estimate of effect; moderate quality, further research is likely to have an important impact on our confidence in the estimate of effect and may change the estimate; low quality, further research is very likely to have an important impact on our confidence in the estimate of effect and is likely to change the estimate; very low quality, we are very uncertain about the estimate.
